# Supplementary material for: Population Pharmacokinetics Modelling and Simulation of Mitotane in Patients with Adrenocortical Carcinoma: An Individualized Dose Regimen to Target All Patients at Three Months?
Source: Pharmaceutics. 2019 Oct 31;11(11):566. doi: 10.3390/pharmaceutics11110566 (PMC6920765; doi:10.3390/pharmaceutics11110566)
Supplement: Supplementary file 1 [file pharmaceutics-11-00566-s001.pdf]

pharmaceutics

Supplementary Materials: Population Pharmacokinetics Modelling and Simulation of Mitotane in Patients with Adrenocortical Carcinoma: An Individualized Dose Regimen to Target All Patients at Three Months?

Yoann Cazaubon, Yohann Talineau, Catherine Feliu, Céline Konecki, Jennifer Russello, Olivier Mathieu, and Zoubir Djerada

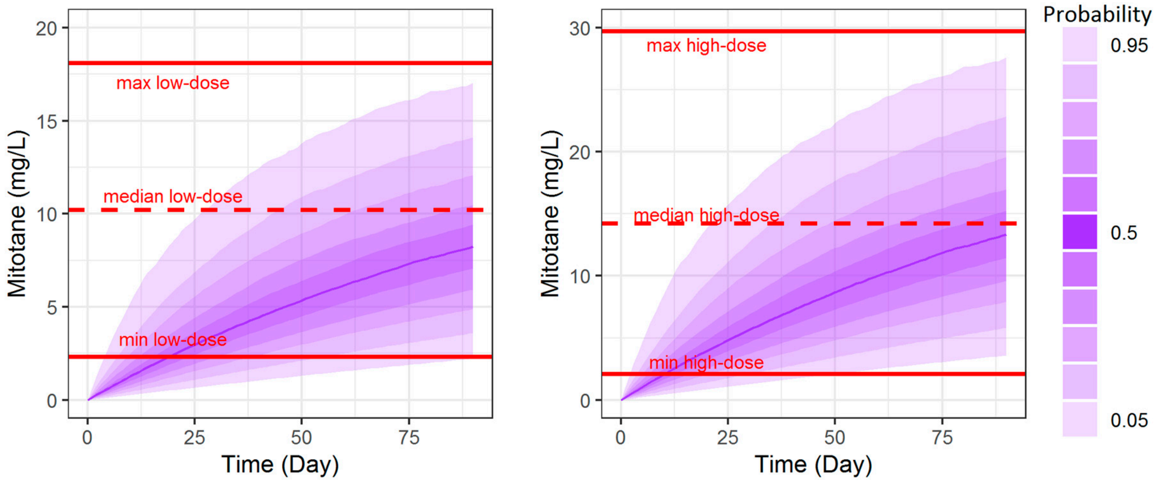

Figure S1. Comparison between simulated concentration profile from our final model versus median (min-max) value of mitotane at 84 days of therapy from study of Kerkhofs et al. [34].

Table S1. Summary of covariates model building and time-varying clearance.

Model	Number of Covariates	−2LL	BIC	ΔBIC	RSE of Parameters
Basic model (1 cmt)	0	3005	3027		< 30%
Tg on Cl (1cmt)	1	2994	3019	−8	< 30%
Tg and HDL on Cl (1cmt)	2	2988	3015	−12	< 30%
Tg, HDL and Lcat2 on Cl (1cmt)	3	2960	2093	−34	< 30%
Basic model with TVC Equation 6	0	2999	3030	−3	> 1000%
Arshad et al.[16]	1	3026	3051	+24	< 30%

Abbreviations are as follows: −2LL = -2 × loglikelihood; ΔBIC = BIC (model step) − BIC (basic model); RSE, Relative standard error; BIC, Bayesian information criterion; lcat2, latent covariate; Tg, triglyceride; TVC, Time-varying clearance.

Equation S1. Summary of equations to model a time-varying clearance.

$$Cl_{linear} = Cl_{initial} + k_{out} \times TIME \quad (1)$$

$$Cl_{exp} = Cl_{initial} \times e^{k_{out} \times TIME} \quad (2)$$

$$Cl_{initial\_exp} = Cl_{initial} + Cl_{ss} \times e^{k_{out} \times TIME} \quad (3)$$

$$Cl_{concave} = Cl_{initial} + Cl_{ss} \times (1 - e^{-k_{out} \times TIME}) \quad (4)$$

$$Cl_{Emax} = Cl_{initial} + Cl_{ss} \times \left(\frac{TIME^{\gamma}}{TIME^{\gamma} + T50^{\gamma}}\right) \quad (5)$$

$$Cl_{pheno1} = Cl_{ss} - (Cl_{ss} - Cl_{initial}) \times \left(\frac{T12}{T12 + TIME}\right) \quad (6)$$

$$Cl_{pheno2} = Cl_{ss} - (Cl_{ss} - Cl_{initial}) \times e^{\left(\frac{-TIME}{T12}\right)} \quad (7)$$

$$Cl_{mecha} = Cl \times \mathrm{ddt}_{\mathrm{Enz}} \left\{= K_{enz} - K_{enz} \times \left(1 - \frac{Cc}{Cc + IC_{50}}\right) \times Enz\right\} \quad (8)$$

Abbreviations are as follows: $Cl_{initial}$ clearance at time = 0, *Clss* induced clearance, k_{out} rate constant for the change in clearance rate. γ gamma (shape factor), *TIME* time after first administration, *T50* time at which clearance of the Cl_{Emax} model reaches 50% of its final value, *T12* time scale at which clearance change, *Cc* mitotane plasma concentration, K_{enz}.
